# Plant-Produced Receptor-Binding Domain of SARS-CoV-2 Elicits Potent Neutralizing Responses in Mice and Non-human Primates

**DOI:** 10.3389/fpls.2021.682953

**Published:** 2021-05-13

**Authors:** Konlavat Siriwattananon, Suwimon Manopwisedjaroen, Balamurugan Shanmugaraj, Kaewta Rattanapisit, Supaporn Phumiamorn, Sompong Sapsutthipas, Sakalin Trisiriwanich, Eakachai Prompetchara, Chutitorn Ketloy, Supranee Buranapraditkun, Wassana Wijagkanalan, Kittipan Tharakhet, Papatsara Kaewpang, Kantinan Leetanasaksakul, Taratorn Kemthong, Nutchanat Suttisan, Suchinda Malaivijitnond, Kiat Ruxrungtham, Arunee Thitithanyanont, Waranyoo Phoolcharoen

**Affiliations:** ^1^Research Unit for Plant-produced Pharmaceuticals, Chulalongkorn University, Bangkok, Thailand; ^2^Department of Pharmacognosy and Pharmaceutical Botany, Faculty of Pharmaceutical Sciences, Chulalongkorn University, Bangkok, Thailand; ^3^Department of Microbiology, Faculty of Science, Mahidol University, Bangkok, Thailand; ^4^BaiyaPhytopharm Co., Ltd., Bangkok, Thailand; ^5^Department of Medical Sciences, Ministry of Public Health, Institute of Biological Products, Nonthaburi, Thailand; ^6^Faculty of Medicine, Center of Excellence in Vaccine Research and Development (Chula Vaccine Research Center, Chula VRC), Chulalongkorn University, Bangkok, Thailand; ^7^Department of Laboratory Medicine, Faculty of Medicine, Chulalongkorn University, Bangkok, Thailand; ^8^Department of Medicine, Faculty of Medicine, Chulalongkorn University, Bangkok, Thailand; ^9^BioNet-Asia Co., Ltd., Bangkok, Thailand; ^10^National Center for Genetic Engineering and Biotechnology (BIOTEC), National Science and Technology Development Agency, Pathum Thani, Thailand; ^11^National Primate Research Center of Thailand-Chulalongkorn University, Saraburi, Thailand

**Keywords:** COVID-19, SARS-CoV-2, receptor-binding domain, *Nicotiana benthamiana*, plant-produced recombinant protein, Fc fusion protein, subunit vaccine

## Abstract

The emergence of coronavirus disease 2019 (COVID-19) caused by severe acute respiratory syndrome coronavirus 2 (SARS-CoV-2) has affected global public health and economy. Despite the substantial efforts, only few vaccines are currently approved and some are in the different stages of clinical trials. As the disease rapidly spreads, an affordable and effective vaccine is urgently needed. In this study, we investigated the immunogenicity of plant-produced receptor-binding domain (RBD) of SARS-CoV-2 in order to use as a subunit vaccine. In this regard, RBD of SARS-CoV-2 was fused with Fc fragment of human IgG1 and transiently expressed in *Nicotiana benthamiana* by agroinfiltration. The plant-produced RBD-Fc fusion protein was purified from the crude extract by using protein A affinity column chromatography. Two intramuscular administration of plant-produced RBD-Fc protein formulated with alum as an adjuvant have elicited high neutralization titers in immunized mice and cynomolgus monkeys. Further it has induced a mixed Th1/Th2 immune responses and vaccine-specific T-lymphocyte responses which was confirmed by interferon-gamma (IFN-γ) enzyme-linked immunospot assay. Altogether, our results demonstrated that the plant-produced SARS-CoV-2 RBD has the potential to be used as an effective vaccine candidate against SARS-CoV-2. To our knowledge, this is the first report demonstrating the immunogenicity of plant-produced SARS-CoV-2 RBD protein in mice and non-human primates.

## Introduction

In December 2019, the unknown pneumonia cases have been first reported in Wuhan, Hubei Province, China, which were initially reported to be caused by novel coronavirus (nCoV-2019) and later named as SARS-CoV-2. The disease condition associated with it is referred as Coronavirus Disease (COVID-19). The virus was closely related to the severe acute respiratory syndrome coronavirus (SARS-CoV) that cause massive outbreak in 2002–2003 (Amanat and Krammer, 2020; Li Q. et al., 2020; Malik et al., 2020; Quinlan et al., 2020; She et al., 2020; Singhal, 2020). In short time, the virus spreads rapidly to more than 200 countries (Malik et al., 2020; She et al., 2020). As of April 2021, more than 130 million confirmed cases with a toll of more than 2.9 million deaths were globally reported and the number of infected patients has still been exponentially increasing daily (World Health Organization, 2021). Furthermore, only few vaccines are currently approved. Thus, the development of affordable effective vaccine or therapeutics is highly essential to control and prevent the infection.

SARS-CoV-2 belongs to the family Coronaviridae in the genera of *Betacoronavirus*, which are known to infect mammals. Coronaviruses (CoVs) are enveloped and single-stranded positive sense RNA viruses (Banerjee et al., 2019; Amanat and Krammer, 2020; Li H. et al., 2020; Rabi et al., 2020; Yuki et al., 2020). The CoV genome consists of 6–11 open reading frames (ORFs) encoding for non-structural polyproteins and structural proteins. The SARS-CoVs have four major structural proteins such as spike (S) surface glycoprotein, membrane (M) protein, envelope (E) protein, and nucleocapsid (N) protein, which are essential for viral assembly and infection (Bosch et al., 2003; Masters, 2006; Banerjee et al., 2019; Li H. et al., 2020; Malla et al., 2020; Rabi et al., 2020; Yuki et al., 2020). S glycoprotein plays a major role in viral attachment to host cells during the viral infection and cleaved by the host proteases into S1 and S2 subunits. SARS-CoV-2 infection starts with pre-fusion of receptor binding domain (RBD) located on the S1 subunit to host receptor, angiotensin converting enzyme 2 (ACE2 receptor) and followed by S2 subunit post-fusion, leading to viral RNA penetration into host cells (Li et al., 2003; Li, 2016; Yuan et al., 2017; Walls et al., 2019; Rabi et al., 2020; Shanmugaraj et al., 2020c; Yuki et al., 2020; Zhou et al., 2020). In addition, RBD was known to elicit potent immune response and considered as prime target for eliciting of host neutralizing antibodies (Wang et al., 2018; Li H. et al., 2020; Smith et al., 2020). Furthermore, previous studies demonstrated that the sera isolated from animals immunized with inactivated SARS-CoV virus significantly neutralize the virus by inhibiting the binding of RBD with ACE2 receptor which proved that the antibodies targeting the RBD domain could prevent the virus infection (He et al., 2004, 2005a,2005b; Zhu et al., 2013; Li H. et al., 2020).

Currently, recombinant proteins are produced mainly by bacterial fermentation or mammalian cell cultures, which still have many limitations including high production costs, immunogenicity profile, and pathogen contamination (Kelley, 2009; Phoolcharoen et al., 2011; Gomes et al., 2016; Kodati et al., 2016; Fuenmayor et al., 2017; Kaur et al., 2018; Rattanapisit et al., 2019a). Plant expression system is considered as the cost-effective platform for the production of vaccine antigens, diagnostic reagents, valuable biopharmaceuticals such as therapeutic immunoglobulins, human enzymes, and human growth factors (Gleba et al., 2005; Miao et al., 2008; Phoolcharoen et al., 2011; Ahmad et al., 2019; Rattanapisit et al., 2019a,b, 2021). Plant expression system offers several advantages in terms of rapidity, flexibility, post-translational modification of recombinant proteins, safety due to lack of animal pathogen, toxin contamination and scalability compared to other available conventional systems (Vitale and Denecke, 1999; Ma et al., 2003; Phoolcharoen et al., 2011; Shahid and Daniell, 2016; Bellucci et al., 2017). Hence, plant platform can be considered as an alternative platform for economical production of commercially viable biopharmaceuticals and vaccines especially for developing countries during pandemic situation (Phoolcharoen et al., 2011; Shanmugaraj et al., 2020a).

In this study, we produced SARS-CoV-2 RBD-Fc fusion protein by fusing SARS-CoV-2 RBD to the Fc domain of human IgG1 at the C-terminus and cloned into geminiviral vector for expression in *Nicotiana benthamiana* plants. The plant-produced RBD-Fc showed specific binding with both human embryonic kidney 293 (HEK293) and Chinese hamster ovary (CHO) cells produced ACE2 protein. Further the plant-produced RBD-Fc was shown to be immunogenic and significantly boosted a humoral and cell-mediated immune response in both mice and cynomolgus macaques (*Macaca fascicularis*). Our results demonstrated that this plant-produced protein has the potential for use as a vaccine candidate against SARS-CoV-2.

## Materials and Methods

### Construction of SARS-CoV-2 RBD-Fc Plant Expression Vector

**FIGURE 1:**
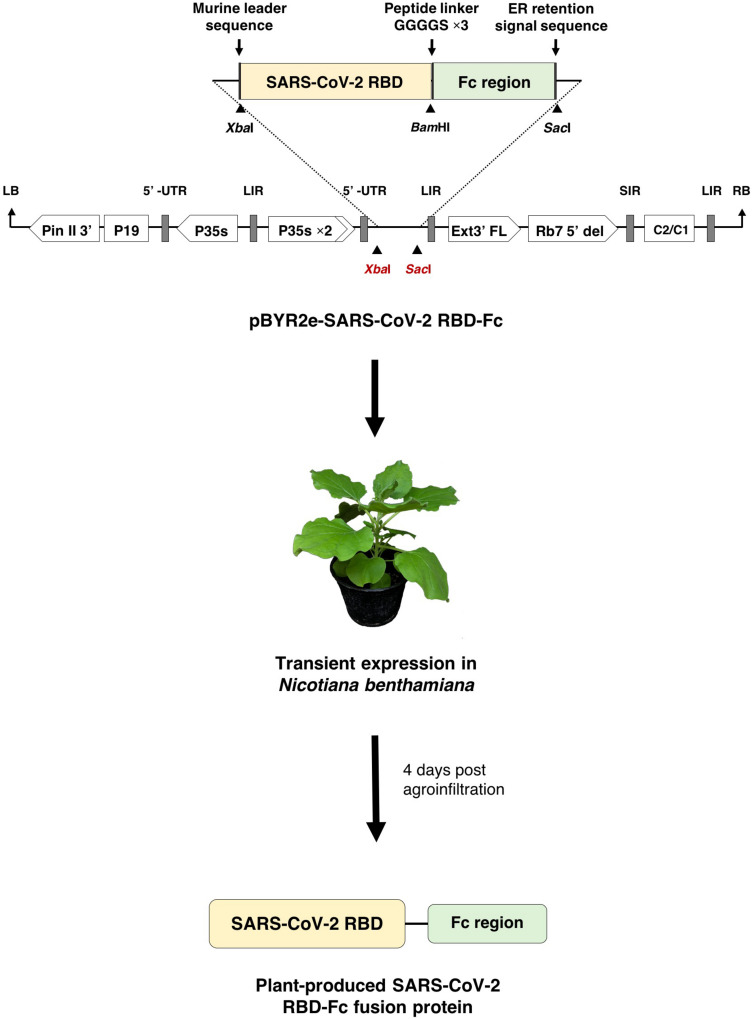
Diagrammatic representation showing the T-DNA of plant expression vector pBYR2e-SARS-CoV-2 RBD-Fc and the overview of transient expression in *N. benthamiana* plants. RB and LB, left and right borders of the T-DNA used in *Agrobacterium* DNA delivery into plant cells; Pin II 3’, the terminator from potato proteinase inhibitor II gene; P19, the RNA silencing suppressor from Tomato Bushy Stunt Virus (TBSV); P35s, 35s promoter from Cauliflower Mosaic Virus (CaMV); P35s × 2, 35s promoter from CaMV with duplicated enhancer; Ext3’ FL, 3’ region of tobacco extension gene; Rb7 5’ del, tobacco RB7 promoter; SIR, short intergenic region of Bean Yellow Dwarf Virus (BeYDV); LIR, long intergenic region of BeYDV; C2/C1, Rep/RepA gene from BeYDV encoding for replication initiation protein (Rep) and RepA.

The RBD of SARS-CoV-2 (SARS-CoV-2 RBD) (Genbank accession number: YP_009724390.1; F318-C617) was designed to anneal with Fc region of human immunoglobulin G1 (IgG1) (GenBank accession number: 4CDH_A) by peptide linker 3XGGGGs at the C-terminus (Figure 1). The nucleotide sequence of SARS-CoV-2 RBD was codon optimized and commercially synthesized (Genewiz, Suzhou, China) with *Xba*I and *Bam*HI restriction sites for cloning with the Fc region, that contains *Bam*HI and *Sac*I restriction sites at the 5′ and 3′ ends, respectively. The SARS-CoV-2 RBD and human Fc region were ligated into a geminiviral vector (pBYR2eK2Md; pBYR2e) (Chen et al., 2011; Diamos and Mason, 2018) using *Xba*I and *Sac*I restriction sites to construct the plant expression vector pBYR2e-SARS-CoV-2 RBD-Fc. The murine leader sequence (Shanmugaraj et al., 2020b) and ER retention signal peptide (SEKDEL) was included at N-terminus and C-terminus of the gene construct, respectively (Figure 1).

### Expression of SARS-CoV-2 RBD-Fc in *Nicotiana benthamiana via*., Agroinfiltration

The plant expression vector pBYR2e-SARS-CoV-2 RBD-Fc was transformed into *Agrobacterium tumefaciens* strain GV3101 cells by electroporation (MicroPulser, Bio-Rad, United States). The recombinant *Agrobacterium* clones were confirmed by polymerase chain reaction (PCR) using the RBD gene-specific primers. *Agrobacterium* containing pBYR2e-SARS-CoV-2 RBD-Fc was resuspended with 1xinfiltration buffer (10 mM 2-(N-morpholino) etanesulfonic acid (MES), 10 mM MgSO4, at pH 5.5) to get final OD_600_ of 0.2 prior to agroinfiltration. The *Agrobacterium* suspension was injected into the adaxial side of 6-week-old *N. benthamiana* leaves. The infiltrated plants were maintained in an optimal 16-h light/8-h dark condition at 28°C and harvested after 4 days post infiltration (dpi).

### Purification of Plant-Produced SARS-CoV-2 RBD-Fc Fusion Protein

The infiltrated leaves were harvested and extracted with 1xPBS (phosphate-buffered saline: 137 mM NaCl, 2.68 mM KCl, 10.1 mM Na_2_HPO_4_, 1.76 mM KH_2_PO_4_ pH 7.4) and clarified by centrifugation at 26,000 *g* for 45 min at 4°C. The supernatant was filtered by using 0.45 μm S-Pak membrane (Merck, Massachusetts, United States). The clarified supernatant was purified by affinity column chromatography with protein-A beads (Expedeon, Cambridge, United Kingdom). The purified column was equilibrated and washed by 1xPBS pH 7.4 followed by elution with 0.1 M glycine buffer pH 3. Subsequently, the pH of the eluted proteins was neutralized by using Tris-HCl pH 8.8. The purified SARS-CoV-2 RBD-Fc was concentrated by using Amicon^®^ ultracentrifugal filter (Merck, Massachusetts, United States) and filtered through 0.22 μm syringe filter (Merck, Massachusetts, United States). The purified plant-produced SARS-CoV-2 RBD-Fc fusion protein was analyzed by using sodium dodecyl sulfate-polyacrylamide gel electrophoresis (SDS-PAGE) and western blotting under reducing and non-reducing conditions. The SARS-CoV-2 RBD-Fc samples were subjected to 8% sodium dodecyl sulfate polyacrylamide gel electrophoresis and stained by Coomassie staining solution for protein visualization. For western blot analysis, the separated proteins were transferred to nitrocellulose membrane (Biorad, United States) and detected with a 1:5,000 sheep anti-human gamma chain-HRP conjugate antibody diluted in 1xPBS (The Binding Site, United Kingdom). The yield of purified plant-produced RBD-Fc fusion protein was estimated by direct ELISA assay using human IgG (Abcam, United Kingdom) as protein standard. The samples were probed by using anti-human gamma chain-HRP fusion (The Binding Site, United Kingdom) at the dilution of 1:1,000 in 1xPBS. A 3,3,5,5′-Tetramethylbenzidine (TMB) solution (Promega, United States) was added into the plate as a colorimetric developer followed by the addition of 1M H_2_SO_4_. The absorbance at 450 nm was read using a 96-well plate reader (Molecular Devices, United States).

### Liquid Chromatography—Mass Spectrometry (LC-MS) of Plant-Produced SARS-CoV-2 RBD-Fc Fusion Protein

The purified proteins were separated on SDS-PAGE. The targeted protein band was excised and sent to National Center for Genetic Engineering and Biotechnology, Pathum Thani, Thailand for LC-MS analysis. The protein was enzymatically digested with trypsin and injected into Hybrid quadrupole Q-Tof impact IITM (Bruker Daltonics Ltd., Hamburg, Germany) equipped with a Nano-captive spray ion source was coupled to a nanoLC system: Ultimate 3000 LC System (Thermo Fisher Scientific, United States). Equipment operation was controlled by Compass 1.9 software (Bruker Daltonics Ltd., Hamburg, Germany). The resulting MS/MS spectra were searched using the Mascot Sever (Matrix Science) against SwissProt database. For Mascot searches, the peptide mass tolerance was set at 0.6 Da and the fragment mass tolerance was set at 1.2 Da.

### ACE2 Binding by ELISA

The binding activity of plant-produced SARS-CoV-2 RBD-Fc fusion protein to ACE2 protein was demonstrated by ELISA. Briefly, 96-well ELISA plate was coated by 100 ng of two different ACE2 protein derived either from HEK293-cells (Abcam, United Kingdom) or CHO-cells (InvivoGen, California, United States). For blocking, 5% skim milk in 1xPBS was added into the wells and incubated for 2 h at 37°C. After blocking, the plate was washed three times with 1xPBST (1xPBS plus 0.05% Tween-20) and incubated with various concentrations of plant-produced SARS-CoV-2 RBD-Fc fusion protein in 1xPBS. The SARS-CoV-2 RBD proteins in the wells were detected by addition of 1: 100 dilution of plant-produced anti-SARS-CoV-2 (H4) mAb (Shanmugaraj et al., 2020b) and followed by a 1:1,000 dilution of anti-human Kappa chain-HRP fusion (SouthernBiotech, United States) in 1xPBS for 1 h at 37°C. For colorimetric development, a TMB solution (Promega, United States) was added into the wells followed by addition of 1M H_2_SO_4_ for terminating the enzymatic reaction. The absorbance at 450 nm was measured using a 96-well microplate reader (Molecular Devices, United States).

### Immunization of Mice and Non-human Primates

Mice immunization protocols were approved by the Institutional Animal Care and Use Committee, Faculty of Medicine, Chulalongkorn University (Protocol review No. 012/2563). Seven-week-old female ICR mice (*n* = 5 per group) were intramuscularly (IM) immunized *via.*, anterior tibialis with 10 μg of plant-produced SARS-CoV-2 RBD-Fc fusion protein without adjuvant or formulated with 0.1 mg alum (InvivoGen, California, United States) on days 0 and 21. Mice sera were collected prior to the first immunization (pre-bleed, day 0) and 14 days post-vaccination to assess the SARS-CoV-2-specific antibody response. The mice were sacrificed on day 35 (14 days after second booster) to collect the spleen for quantitative measurement of SARS-CoV-2 RBD-specific T-cell responses.

For non-human primate immunogenicity studies, all procedures were reviewed and approved by the National Primate Research Center of Thailand-Chulalongkorn University (NPRCT-CU) Animal Care and Use Committee (Protocol review No. 207512) and the facility has been AAALAC International Accredited (1752). Thirteen SPF juvenile cynomolgus macaques (*Macaca fascicularis*), aged between 2.5 and 3.5 years old, and body weight between 2.18 and 3.17 kg, were assigned into three groups in which the control group (*n* = 3) was immunized by PBS adjuvanted with 0.5 mg alum, and other two groups (*n* = 5 per group) were administered with 25 and 50 μg of plant-produced SARS-CoV-2 RBD-Fc fusion protein along with 0.5 mg alum adjuvant, respectively. Monkeys were received two intramuscular injections on day 0 and 21. The blood samples were collected on day 0 (pre-immunization) and 14 days after each vaccination (days 14 and 35) to assess SARS-CoV-2 RBD specific IgG, neutralizing antibody and cell-mediated immune responses.

### Evaluation of SARS-CoV-2 RBD-Specific Total Antibody Responses by ELISA

96-well plate was coated with 100 ng of SARS-CoV-2 spike protein RBD derived from Sf9 insect cells (GenScript, United States) and incubated overnight at 4°C. Then, the wells were blocked with 5% skim milk powder in 1xPBS for 2 h at 37°C. Subsequently, the animal sera were twofold serially diluted with 1xPBS starting at 1:100 was loaded on the wells and incubated for 2 h at 37°C. Goat anti-mouse IgG HRP conjugate antibody (Jackson ImmunoResearch, Pennsylvania, United States) and goat anti-monkey IgG HRP conjugation (Abcam, United Kingdom) diluted 1:2,000 in 1xPBS were added into the wells for detecting mouse and monkey antibodies, respectively, and the plate was incubated for 2 h at 37°C. TMB substrate (Promega, United States) was added into the plates for colorimetric development. The enzymatic reactions were terminated by adding 1M H_2_SO_4_. The absorbance was measured at 450 nm using a microplate reader (BMG Labtech, Germany). Between each step, the plates were washed by 1xPBST for three times. For mouse IgG1 and IgG2a analysis, the mice sera were twofold serially diluted with 1xPBS starting 1:100 in the same fashion and detected by 1:2,000 goat anti-mouse IgG1 (HRP) and goat anti-mouse IgG2a heavy chain (HRP) antibody (Abcam, United Kingdom), respectively, diluted in 1xPBS. The endpoint titer of IgG1 and IgG2a were also computed for monitoring Th2 and Th1 lymphocyte responses, respectively. The endpoint titers were determined as the highest dilution of immunized sera, which had A_450_ more than cut off calculated from A_450_ of pre-immunized sera in the dilution of 1:100 in 1xPBS (Frey et al., 1998). All experiments were performed in duplicates and 1xPBS was used as a control. The statistical analyses of immunological data were performed using GraphPad Prism software version 8.0. Statistical significance was calculated by two-way analysis of variance (ANOVA). All data in each group were compared by using Tukey’s multiple comparisons test and the values of *p* < 0.05 were considered as statistically significant.

### *In vitro* Microneutralization Assay

Microneutralization assay was performed using Vero E6 cell line and live SARS-CoV-2 virus isolated from a COVID-19 patient and conducted in a certified biosafety level 3 facility of Microbiology department, Faculty of Science, Mahidol University, Bangkok, Thailand. The cells were plated in 96-well plate at 1 × 10^4^ cells/well in DMEM (Dulbecco’s Modified Eagle’s medium supplemented with 10% heat-inactivated FBS, 100 U/mL of penicillin and 0.1 mg/mL of streptomycin) and incubated for overnight. The immunized sera and positive convalescent serum from COVID-19 patient were heat-inactivated at 56°C for 30 min. The immunized sera were twofold serially diluted in duplicates and incubated with 100 of 50% tissue culture infective dose (TCID_50_) of the SARS-CoV-2 virus in DMEM at 37°C for 1 h. Virus control at 100TCID_50_ and uninfected cell control wells were included in all plates. Subsequently, the mixture of diluted serum and virus was transferred to the cell monolayer and incubated at 37°C with 5% CO_2_ for 2 days. After incubation, the cells were washed once with 1xPBS and then fixed and permeabilized with ice-cold 1:1 methanol/acetone fixative solution at 4°C for 20 min. The cells were washed 3 times with 1xPBST and blocked with 2% BSA at room temperature (RT) for 1 h. After washing, the viral nucleocapsid was detected using 1:5,000 of SARS-CoV/SARS-CoV-2 nucleocapsid (N) monoclonal antibody (SinoBiological, United States) and incubated at 37°C for 1 h followed by adding 1:2,000 of HRP-conjugated goat anti-rabbit polyclonal antibody (Dako, Denmark) diluted with 1xPBS and incubated at 37°C for 1h. The KPL Sureblue^TM^ TMB substrate (SeraCare, United States) was added for colorimetric development. Then the reaction was stopped by the addition of 1N HCl. The absorbance at 450 and 620 nm was read by a Sunrise^TM^ microplate reader (Tecan, Switzerland). The absorbance differences between 450 and 620 nm (A_450_–A_620_) of diluted samples were compared with the 50% specific signal of the cut point, which was calculated by the following equation to determine the potent neutralization titers of the immunized sera.

$$A_{c\,u\,t\,p\,o\,i\,n\,t}=\left(\frac{A_{v\,i\,r\,u\,s\,c\,o\,n\,t\,r\,o\,l}-A_{c\,e\,l\,l\,c\,o\,n\,t\,r\,o\,l}}{2}\right)+A_{c\,e\,l\,l\,c\,o\,n\,t\,r\,o\,l}$$

Where *A*_*Virus control*_ and *A*_*cell control*_ are the average of A_450_-A_620_ of virus control wells and cell control wells, respectively. The neutralizing titers were defined as the reciprocal highest dilution providing the average of A_450_–A_620_ of the diluted serum well more than the cut point. The neutralizing antibody titers of each experimental group were compared by GraphPad Prism 8.0 using Mann-Whitney test. The significant differences were considered when *p* < 0.05.

### Mouse IFN-γ ELISPOT Assay

For mouse splenocyte preparation, the spleen cells were aseptically plated in the petri-dish and dissociated into single-cell suspension using needle#21 for 2–3 times. The separated splenocytes were cultured in R5 medium (RPMI1640 with 100 U/mL penicillin, 100 U/mL streptomycin, 5% heat-inactivated fetal bovine serum (FBS, Gibco, United States) and 2-mercaptoethanol) and centrifuged at 1,200 g 4°C for 5 min. Subsequently, splenocytes were lysed with 1xACK lysis buffer, added R5 medium and collected the cell pellet by centrifugation at 1,200 g 4°C for 5 min. Finally, splenocytes were counted and adjusted for using in ELISpot assay. The IFN-γ secreting cells were quantified by using mouse IFN-γ ELISpot assay kit (Mabtech, Stockholm, Sweden). Briefly, splenocytes were resuspended at 5 × 10^6^ cells/mL in R5 medium. The 96-well plates (Millipore, Bedford, MA, United States) were coated with 500 ng of anti-mouse IFN-γ (AN18) monoclonal antibody (mAb) (Mabtech, Stockholm, Sweden) in 1xPBS at 37°C with 5% CO_2_ for 3 h. Then, the plates were washed six times with 1xPBS and blocked with R10 medium at RT for 1 h. A quantity of 5 × 10^5^ splenocytes per well were activated by lyophilized SARS-CoV-2 peptide pools (BioNet-Asia, Thailand, and Mimotopes, Australia) at a final concentration of 2 μg/mL at 37°C with 5% CO_2_ for 40 h. R5 medium and concanavalin A (ConA) were used as negative and positive controls, respectively. After incubation, the plates were washed six times with 1xPBST followed by three times with 1xPBS. Then, the plates were incubated with anti-mouse IFN-γ-biotinylated mAb (Mabtech, Stockholm, Sweden) diluted in 1xPBS at RT for 3 h. After washing, streptavidin-alkaline phosphatase (ALP: Mabtech, Stockholm, Sweden) was added and incubated at RT for 1 h. The substrate solution (5-bromo-4-chloro-3-indolyl-phosphate/nitro blue tetrazolium; BCIP/NBT) were added into the wells and incubated until distinct spots emerge. Reactions were stopped by washing extensively in tap water and rinsing the underside of membrane. Inspect and count spots were performed with an ELISpot reader (ImmunoSpot^®^ Analyzer, United States). Results were expressed as spot-forming cells (SFCs)/10^6^ splenocytes. The positive responses were defined as > 50 SFCs/10^6^ splenocytes over the background signal. The result analyses were conducted using Kruskal-Wallis test in GraphPad Prism 6.0. All *p*-values < 0.05 were defined as significant.

### Non-human Primate IFN-γ ELISpot Assay

For peripheral blood mononuclear cells (PBMCs) preparation, the cells were isolated by density gradient separation using Isoprep (Robbins Scientific Corporation, CA). Briefly, after removal of plasma, EDTA-treated whole blood was diluted (1:1) with RPMI1640 medium containing 2 mM L-Glutamine (Gibco, United States) and layered over Isoprep. Samples were then centrifuged at 1,200 g for 30 min. The PBMC layer was harvested and washed twice with RPMI1640. Then, the cells were resuspended in R10 medium (RPMI1640 supplemented with 100 U/mL of penicillin, 100 U/mL of streptomycin and 10% heat-inactivated fetal bovine serum (FBS, Gibco, United States) for applying in ELISpot assay. The antigen-specific cells secreting monkey IFN-γ were enumerated by using monkey IFN-γ ELISpot assay kit (Mabtech, Stockholm, Sweden). Briefly, PBMCs were stimulated with SARS-CoV-2 spike peptides pools (BioNet-Asia, Thailand, and Mimotopes, Australia) at 37°C with 5% CO_2_ for 40 h. R5 medium and phytohemagglutinin (PHA) were served as negative and positive control, respectively. The secreted monkey IFN-γ were detected by anti-monkey IFN-γ-biotinylated mAb (Mabtech, Stockholm, Sweden) and followed by addition of ALP solution (Mabtech, Stockholm, Sweden). For spot development, BCIP/NBT-plus substrate solution was added into the wells and incubated until distinct spots emerge. The spots were inspected and counted by ELISpot reader (ImmunoSpot^®^ Analyzer, Germany). Results were expressed as spot-forming cells (SFCs)/10^6^ PBMCs following the subtraction of the negative control. The positive responses were defined > 100 SFCs/10^6^ PBMCs over the background. Statistical analyses were presented using GraphPad Prism 8.0. Comparison of frequencies of populations in each group was made using Friedman and Mann-Whitney tests. All *p-*values < 0.05 were defined as significant.

## Results

### Transient Expression of SARS-CoV-2 RBD-Fc Fusion Protein in *Nicotiana benthamiana*

**FIGURE 2:**
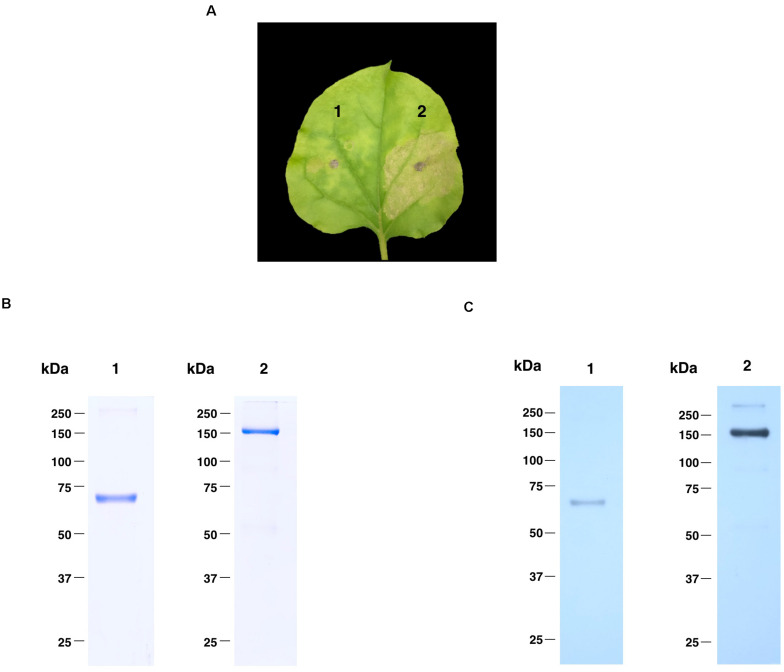
Expression profiles of plant-produced SARS-CoV-2 RBD-Fc fusion protein. Phenotype of leaf infiltrated with *Agrobacterium* control (1) and *Agrobacterium* containing pBYR2e-SARS-CoV-2 RBD-Fc (2) after 4 dpi **(A)**. SDS-PAGE analysis of plant-produced SARS-CoV-2 RBD-Fc fusion protein stained with Coomassie staining **(B)** and western blot of plant-produced SARS-CoV-2 RBD-Fc fusion protein probed with anti-human gamma-HRP conjugate antibody **(C)**. Lane 1 and 2, purified plant-produced SARS-CoV-2 RBD-Fc fusion protein under reducing and non-reducing condition, respectively.

The nucleotide sequence of RBD of SARS-CoV-2 was codon-optimized and fused with Fc region of human IgG1 at the C terminus of the RBD gene construct. The codon-optimized SARS-CoV-2 RBD-Fc fusion gene was cloned into the geminiviral plant expression vector pBYR2e. For expression of RBD-Fc fusion protein in plants, *N. benthamiana* plants were infiltrated with *Agrobacterium* containing pBYR2e-SARS-CoV-2 RBD-Fc (Figure 1). The leaves infiltrated with *Agrobacterium* containing pBYR2e-SARS-CoV-2 RBD-Fc showed significant phenotypic necrosis compared to the leaves infiltrated by *Agrobacterium* without the plant expression vector (Figure 2A).

### Purification and Characterization of Plant-Produced RBD-Fc Fusion Protein

Plant-produced SARS-CoV-2 RBD-Fc fusion protein was extracted and purified from crude extract by single-step protein A affinity chromatography. The purified SARS-CoV-2 RBD-Fc fusion protein was concentrated and filtered by using 0.22 μm syringe filter. The purity of the purified plant-produced SARS-CoV-2 RBD-Fc was analyzed by SDS-PAGE gel stained with Coomassie blue staining under reducing and non-reducing condition. The purity of SARS-CoV-2 RBD-Fc fusion protein was found to be higher than 90% based on the visual inspection of a Coomassie-stained SDS gel (Figure 2B). The SARS-CoV-2 RBD-Fc fusion protein was further analyzed by western blot probed with anti-human gamma chain-HRP conjugate antibody. The protein band corresponding to the molecular weight of 65 kDa was observed in reducing condition (Figure 2C; lane 1). In addition, the plant-produced SARS-CoV-2 RBD-Fc fusion protein under non-reducing condition was observed at approximately 150 kDa (Figure 2C; lane 2), which implies that the SARS-CoV-2 RBD-Fc fusion protein could be linked by disulfide bond into dimeric form. The expression level of plant-produced SARS-CoV-2 RBD-Fc was quantified by ELISA and found to be 25 μg/g fresh weight. The authenticity of purified plant-produced SARS-CoV-2 RBD-Fc fusion protein was further confirmed by using a high-resolution LC-TOF MS/MS analysis as shown in Supplementary Document.

### *In vitro* Binding Activity of Plant-Produced RBD-Fc Fusion Protein

**FIGURE 3:**
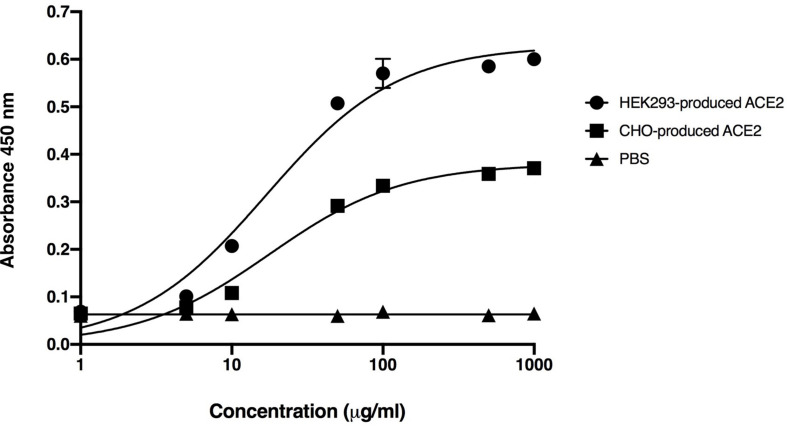
Binding activity of the plant-produced SARS-CoV-2 RBD-Fc with the commercial angiotensin-converting enzyme 2 (ACE2 proteins) derived from HEK293 and CHO cells analyzed by ELISA. PBS was used as negative control. Data are presented as mean ± standard deviation (SD) of triplicates in each sample dilution.

The binding of plant-produced SARS-CoV-2 RBD-Fc fusion protein was confirmed by ELISA by using commercial HEK293 and CHO-produced ACE2 protein as the capture reagent. The various dilutions of purified plant-produced SARS-CoV-2 RBD-Fc was incubated with commercial ACE2 proteins. For detection of SARS-CoV-2 RBD, anti-SARS-CoV-2 (H4) mAb (Shanmugaraj et al., 2020b) and anti-human kappa chain-HRP conjugate antibody were added into the wells. The results showed that plant-produced SARS-CoV-2 RBD-Fc fusion protein produced saturable binding to both commercial ACE2 proteins with substantially high affinity in comparison to PBS control (Figure 3), which confirms the authenticity of plant-produced SARS-CoV-2 RBD-Fc.

### Immunogenicity in Mice

**FIGURE 4:**
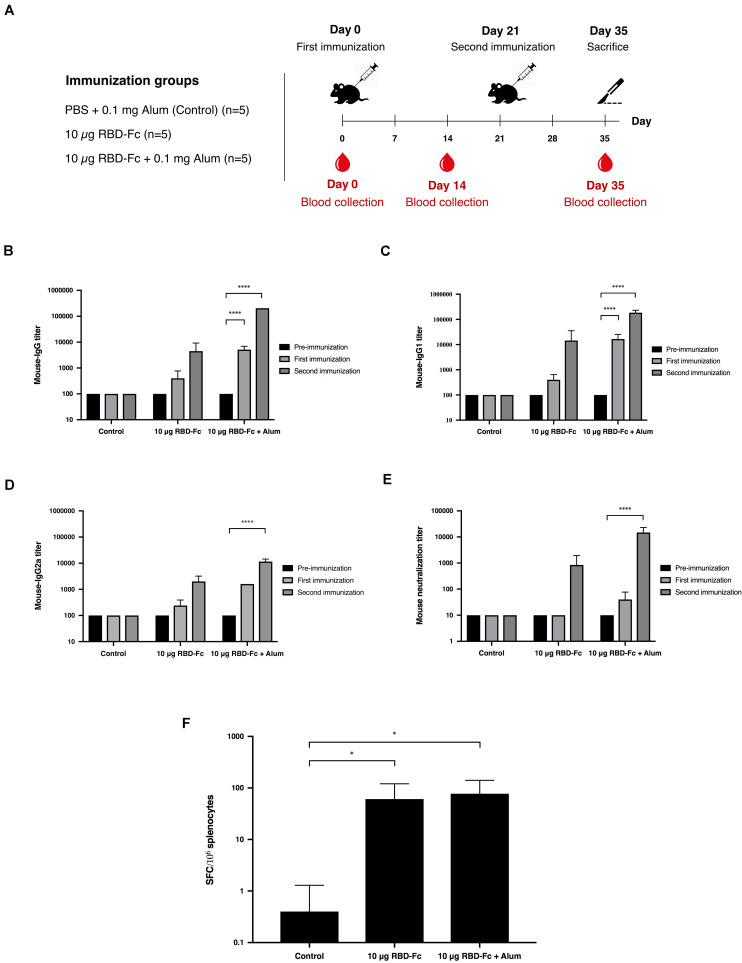
Immunogenic studies in mice. Schematic representation of immunization protocol and sample collection. Groups of mice (five mice per each group) were intramuscularly immunized with 10 μg of SARS-CoV-2 RBD-Fc fusion protein alone or with alum adjuvant, followed by booster dose at 21 days after first immunization. Mice sera were collected on day 0 (pre-bleed) and day 14 post-immunization **(A)**. Titers of SARS-CoV-2 RBD-specific total IgG **(B)**, IgG1 **(C)**, and IgG2a **(D)** in the immunized sera collected on day 0, 14, and 35 were analyzed by indirect ELISA using Sf9-produced SARS-CoV-2 RBD-His as the capture antigen. Potent neutralizing antibody titers in mice sera were tested by *in vitro* microneutralization assay using Vero E6 cell line and live SARS-CoV-2 **(E)**. The functional profiles of SARS-CoV-2 RBD-specific T-cell responses expressing in mouse splenocytes immunized with plant-produced SARS-CoV-2 RBD-Fc adjuvanted with alum were determined by mouse ELISpot assay **(F)**. Data presented as mean ± SD of the endpoint titers in each mice vaccination group (*n* = 5). ^∗^*p* < 0.05; ^∗∗^*p* < 0.01; ^∗∗∗^*p* < 0.001; ^∗∗∗∗^*p* < 0.0001.

Mice immunogenicity was assessed in 7-week-old female Mlac:ICR mice by immunizing intramuscularly on days 0 and 21 with 10 μg of plant-produced SARS-CoV-2 RBD-Fc fusion protein with or without alum adjuvant. Mice sera were collected on days 0, 14, and 35 (Figure 4A). SARS-CoV-2 RBD-specific antibodies were evaluated by ELISA using commercial SARS-CoV-2 RBD-His protein produced from Sf9 cells as a capture antigen. The SARS-CoV-2 RBD-specific immune responses were observed after first immunization of plant-produced SARS-CoV-2 RBD-Fc alone, whilst a slightly increased specific-mouse total IgG response was observed in SARS-CoV-2 RBD-Fc immunized with alum. All mice immunized with plant-produced RBD-Fc elicited significantly higher antibody titer after second booster compared with the control group (Figure 4B). Plant-produced SARS-CoV-2 RBD-Fc was found to be immunogenic, while the addition of alum adjuvant could significantly improve its immunogenicity. In addition, we appraised the levels of SARS-CoV-2 RBD-specific IgG1 and IgG2a subclasses, which are indicators of Th2 and Th1 lymphocyte responses in mice, respectively. The results demonstrated that plant-produced SARS-CoV-2 RBD-Fc induced both SARS-CoV-2 RBD-specific IgG1 (Figure 4C) and IgG2a (Figure 4D) with the IgG1 bias. The *in vitro* neutralizing ability of the immunized sera was evaluated. The SARS-CoV-2 RBD-Fc without adjuvant induced neutralizing antibody against SARS-CoV-2 after the second dose at a dilution of 1:1,280 (Figure 4E). Interestingly, mice sera immunized by SARS-CoV-2 RBD-Fc adjuvanted with alum displayed maximum SARS-CoV-2 neutralization with a dilution more than 1:10,240 (Figure 4E). IFN-γ levels of splenocytes isolated from mice was tested by IFN-γ ELISpot assay. The results showed that plant-produced RBD-Fc elicited IFN-γ secretion which was statistically significant compared with mock control group (Figure 4F).

### Immunogenicity in Non-human Primates

**FIGURE 5:**
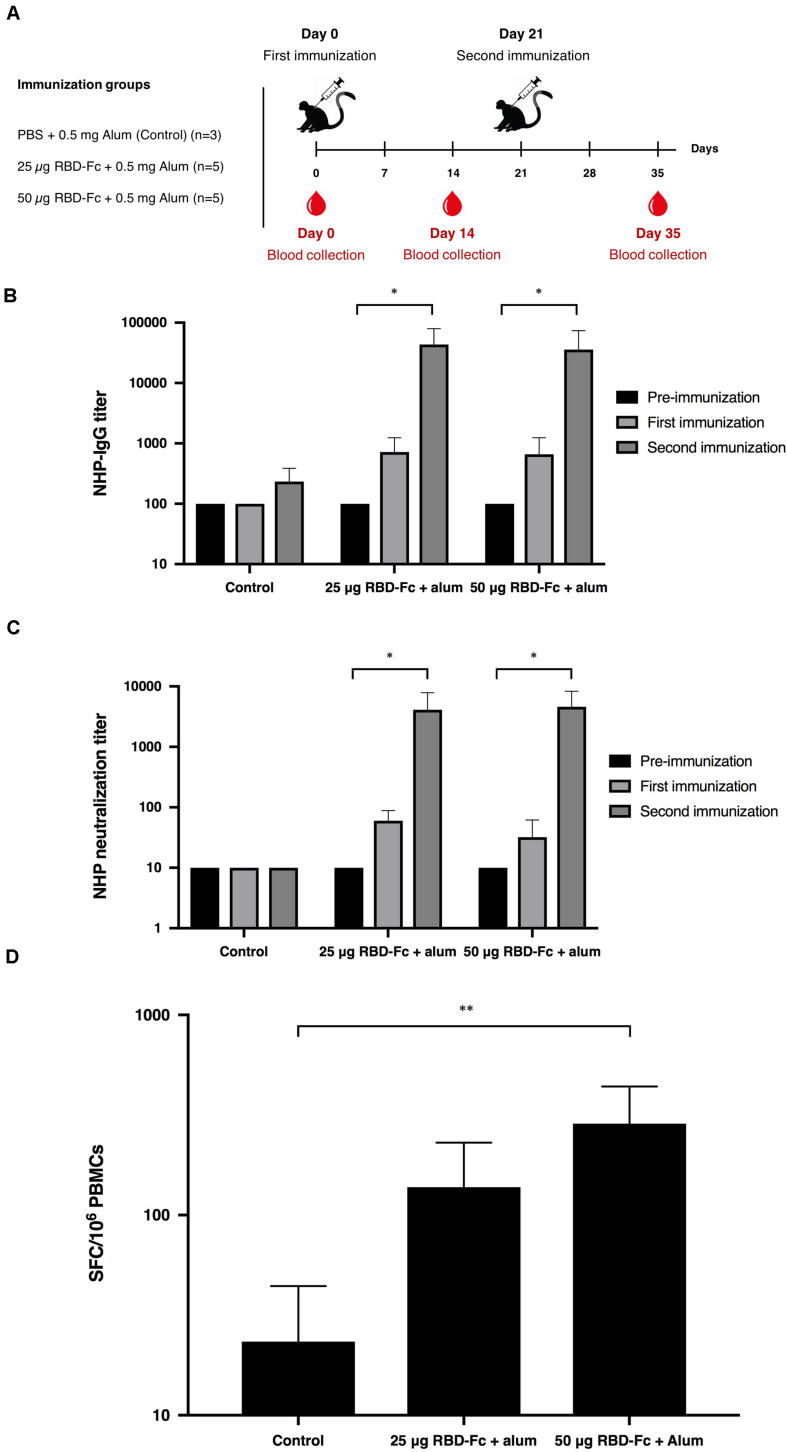
Immunogenic studies in non-human primates (*Macaca fascicularis*). Experimental design of immunogenicity studies in non-human primates. Thirteen juvenile-adult non-human primates were separated into 3 groups; Control group was immunized with PBS adjuvanted by alum (*n* = 3) and two experimental groups were immunized with 25 and 50 μg SARS-CoV-2 RBD-Fc along with alum adjuvant (*n* = 5 per group). All non-human primates were intramuscularly injected either with PBS or plant-produced RBD-Fc for 3 weeks interval (on day 0 and 21). The sera were collected on day 0 and 14 after each boost **(A)**. Serum specific IgG response in non-human primates were determined by ELISA **(B)**. Virus neutralizing titer of RBD immunized non-human primate sera against live SARS-CoV-2 were evaluated **(C)**. The functional profiles of SARS-CoV-2 RBD-specific T-cell responses expressing in non-human primate peripheral blood mononuclear cells immunized with plant-produced RBD-Fc adjuvanted with alum on day 14 after second immunization **(D)**. ^∗^*p* < 0.05; ^∗∗^*p* < 0.01; ^∗∗∗^*p* < 0.001; ^∗∗∗∗^*p* < 0.0001.

Cynomolgus macaques (*Macaca fascicularis*) were intramuscularly immunized with 25 and 50 μg of plant-produced SARS-CoV-2 RBD-Fc with the presence of alum on day 0 and 21. Monkey sera were collected on day 0, 14, and 35 (Figure 5A). Plant-produced SARS-CoV-2 RBD-Fc protein adjuvanted with alum was capable of inducing dose-independent SARS-CoV-2 RBD-specific IgG antibodies in monkeys after first and second immunization with the dilution 1:800, and 1:51,200, respectively (Figure 5B). Specifically, the microneutralization assay was performed by using Vero E6 cell line to evaluate the level of neutralizing antibodies against live SARS-CoV-2. Sera collected 14 days after the first immunization (day 14) showed neutralizing activity against the SARS-CoV-2 and increased at day 35 (14 days after the second immunization) with the neutralization titer approximately 5,120 (Figure 5C). In addition, cell-mediated immune responses in cynomolgus monkeys were monitored by IFN-γ ELISpot assay by using peripheral blood mononuclear cells isolated from immunized sera on day 35. The specific IFN-γ expression was detected in monkeys immunized with two doses of plant-produced SARS-CoV-2 RBD-Fc, and a significant difference from the control group was detected compared to immunized monkeys (Figure 5D).

## Discussion

The recent emergence of coronavirus diseases (COVID-19) in China is responsible for the current global pandemic and public health crisis (Li Q. et al., 2020; Malik et al., 2020; Singhal, 2020; Yuki et al., 2020). Few vaccines are currently approved for human use. Hence, it is highly essential to develop safe, effective vaccines and therapeutics against this infection to prevent its spread. Recently, several groups have predicted and assessed the immunogenicity potential of SARS-CoV-2 related proteins and showed that SARS-CoV-2 S protein is the suitable candidate for recombinant vaccine development as it can elicit potent immune response and is the major target of neutralizing antibodies. The receptor-binding domain (RBD) of SARS-CoV-2 plays a key role in viral attachment and entry into the host cells by interaction with the ACE2 receptor in the host cells (Li et al., 2003; Lei et al., 2020; Quinlan et al., 2020; Rabi et al., 2020; Shanmugaraj et al., 2020c; Sun et al., 2020; Xie et al., 2020; Yuki et al., 2020). Particularly, RBD domain has multiple conformational epitopes, which can induce host immune responses and highly potent neutralizing antibodies (He et al., 2005a,b; Zhu et al., 2013; Wang et al., 2018; Smith et al., 2020). Hence, RBD domain in the S protein is considered as a potential target and could serve as a potent immunogen for developing the possible SARS-CoV-2 vaccines.

Since 1980s, plants have been utilized for the development of highly valuable biopharmaceuticals either for human or veterinary applications. Plant-based expression system offers several key advantages over conventional systems in terms of production speed, cost, and safety (Basaran and Rodríguez-Cerezo, 2008; Paul and Ma, 2011; Krenek et al., 2015; Yao et al., 2015; Burnett and Burnett, 2019; Shanmugaraj et al., 2020a; Daniell et al., 2021). Remarkably, plant-based expression system can produce large amounts of recombinant antigens in a relatively short time period within few weeks after making the gene construct (D’Aoust et al., 2008, 2010; Rybicki, 2009; Vézina et al., 2009; Pillet et al., 2016; Rattanapisit et al., 2020; Ward et al., 2020). The concept of plant-produced biopharmaceuticals and vaccines has been assessed and well explored by number of research groups worldwide. Several plant-produced therapeutics (Kizhner et al., 2015; Ma et al., 2015; Mor, 2015; Prevail Ii Writing Group et al., 2016) and vaccines (Yuki et al., 2013; Hendin et al., 2017; Donini and Marusic, 2019) are currently in pipeline for clinical trials and approval, Notably plant-produced Glucocerebrosidase enzyme (Elelyso^TM^) for the treatment of type I Gaucher’s disease has been approved by US Food and Drug Administration (Fox, 2012) and tobacco-derived seasonal flu VLP vaccine from Medicago Inc., is currently in final stages of clinical trial (Ward et al., 2021). Hence, plant-based expression could be an alternative option for rapid production of emergency vaccines or therapeutic antibodies (Iyappan et al., 2018; Diego-Martin et al., 2020; Rattanapisit et al., 2020; Shanmugaraj and Phoolcharoen, 2021; Siriwattananon et al., 2021).

The geographical distribution of virus is increasing rapidly and global concern on this pandemic demands an affordable and scalable protein production platform that can produce recombinant proteins relatively in short time with much reduced cost. Hence in this study, we have demonstrated the rapid production of SARS-CoV-2 RBD-Fc fusion protein in *N. benthamiana* plants that could be used as a potential vaccine candidate for prevention of SARS-CoV-2 infection.

Significant efforts have been made by the scientific community across the world to develop the effective vaccine against SARS-CoV-2. Plant-derived vaccine candidates for other coronaviruses such as SARS and porcine epidemic diarrhea virus are shown to elicit potent immunogenic response in animal studies (Kang et al., 2005; Pogrebnyak et al., 2005). Earlier studies showed that full length S protein-based vaccine could cause liver damage or enhance virus infection. RBD-based vaccines formulated with alum was shown to elicit high level of protective immunity in animal experiments (He et al., 2006; Du et al., 2007; Jiang et al., 2012; Chen et al., 2017, 2020a,2020b). Hence based on the available data on the immunogenicity of SARS-CoV-2 proteins, we have chosen RBD for plant expression.

The presence of Fc domain in fusion protein offers favorable characteristics such as improving the expression and secretion of the recombinant proteins, improving protein solubility and stability (Huang, 2009; Czajkowsky et al., 2012; Yang et al., 2018). Moreover, Fc domain increases the serum half-life and prolongs therapeutic protein activities due to pH-dependent binding to the neonatal Fc receptor (FcRn) leading to prevention of protein degradation in endosomes as well as reduces renal clearance rate due to larger molecular weight of protein (Jazayeri and Carroll, 2008; Suzuki et al., 2010; Carter, 2011; Rath et al., 2015; Rosales-Mendoza et al., 2017; Yang et al., 2018). Fc region have been used as a fusion protein partner for several recombinant proteins such as receptors, ligands, enzymes, and soluble cytokines for therapeutic applications (Strohl and Knight, 2009; Li and Ravetch, 2011; Czajkowsky et al., 2012; Rattanapisit et al., 2019c; Liu et al., 2020). In addition to the mentioned advantages, Fc region is used as a tagged protein for facilitating the effective purification of recombinant protein by protein A chromatography that can provide high purity of SARS-CoV-2 RBD-Fc which can be visualized on the Coomassie-stained SDS-PAGE compared to crude extract sample as shown in previous studies (Rattanapisit et al., 2019c; Siriwattananon et al., 2021). Hence, we engineered SARS-CoV-2 RBD by fusing with Fc region of human IgG1 in order to use it as a subunit vaccine against SARS-CoV-2.

The SARS-CoV-2 RBD-Fc fusion protein was transiently expressed as a soluble protein in plants. The results showed that the expression of SARS-CoV-2 RBD-Fc was achieved rapidly within 4 days post infiltration with necrosis signal was observed on the infiltrated leaves (Figure 2A). The recombinant protein was purified from the plant crude extracts by affinity column chromatography and used for further studies.

The plant-produced SARS-CoV-2 RBD-Fc apparently showed effective binding activity with commercial ACE2 proteins produced from HEK293 and CHO cells (Figure 3). This data indicated that the SARS-CoV-2 RBD protein folded correctly in plants and produced authentic antigen. The immunogenicity of plant-produced SARS-CoV-2 RBD-Fc was tested in mice and cynomolgus monkeys using alum as an adjuvant. Alum stimulates the innate immunity, particularly presenting the antigen to major histocompatibility complex (MHC) class II, CD40 and CD86 or inducing the Th2 responses to mediate B-cell differentiation and elicit the antigen specific-IgG1 isotype (Marrack et al., 2009; Exley et al., 2010; Zhang et al., 2016). Furthermore, alum is having good safety profile and has been used as an adjuvant in several currently available licensed vaccines to enhance the immune response of the antigen (HogenEsch et al., 2018).

Mice administered with two doses of plant-derived SARS-CoV-2 RBD-Fc protein formulated with alum as adjuvant developed the neutralizing immune response (Figure 4E). The results confirmed the immunogenicity of plant-produced recombinant SARS-CoV-2 RBD protein. Mice immunized with RBD with alum showed the higher titer of neutralizing antibodies compared with the mice immunized with SARS-CoV-2 RBD alone. The analysis of the mouse specific-IgG subtypes suggested that plant-produced SARS-CoV-2 RBD induced a mixed Th1/Th2-specific immune responses. Further the efficacy of plant-produced SARS-CoV-2 RBD-Fc fusion protein was investigated in cynomolgus monkeys by administering the SARS-CoV-2 RBD-Fc with alum as adjuvant. The results confirmed that the plant-produced SARS-CoV-2 RBD-Fc could induce neutralizing antibodies in monkeys (Figure 5C). To accomplish the capability of plant-produced SARS-CoV-2 RBD-Fc in induction of cell-mediated immune responses, mouse splenocytes and monkey peripheral blood mononuclear cells were collected 14 days after second immunization (Figures 4F, 5D). The IFN-γ-expressing T cells were analyzed by ELISPOT assay. Plant-produced SARS-CoV-2 RBD-Fc without alum induced SARS-CoV-2-specific T-cell responses, as evidenced by significant IFN-γ expression compared with the control (Figure 4F). Addition of alum adjuvant did not significantly increase the number of IFN-γ in the animals. These results suggested that plant-produced SARS-CoV-2 RBD-Fc itself could induce T-cell responses.

These results clearly showed that the plant-expressed SARS-CoV-2 RBD-Fc fusion protein maintains their authentic structure and retains its antigenicity. Our results are consistent with those of previous studies which showed that the vaccine antigens expressed in *N. benthamiana* elicited potent immune responses in animal experiments (Zheng et al., 2009; He et al., 2014). In consistent with earlier reports on the immunogenicity of RBD of SARS-CoV, our study showed that neutralizing antibodies induced by plant produced RBD of SARS-CoV-2 suppress SARS-CoV-2 infection *in vitro* (Bisht et al., 2005; Kapadia et al., 2005; Du et al., 2010). Our data showed the potential of plant-produced subunit vaccine candidate as the effective SARS-CoV-2 vaccine.

## Conclusion

In conclusion, our study demonstrated that it was feasible to produce SARS-CoV-2 RBD protein in *N. benthamiana* plants by transient expression system. Further plant-produced recombinant protein was shown to be immunogenic in mice and non-human primates. The vaccine elicited both humoral and cell mediated immune responses suggesting the potential of plant- produced RBD as the effective vaccine against SARS-CoV-2. To our knowledge, this is the first report demonstrating the immunogenicity of plant-produced SARS-CoV-2 RBD protein in mice and non-human primates. Collectively this proof of concept study demonstrated that the plant-produced SARS-CoV-2 proteins could possibly be further developed as candidate vaccines for early stage clinical development.

## Data Availability Statement

The original contributions presented in the study are included in the article/Supplementary Material, further inquiries can be directed to the corresponding author/s.

## Ethics Statement

The animal study was reviewed and approved by the Institutional Animal Care and Use Committee, Faculty of Medicine and the National Primate Research Center of Thailand-Chulalongkorn University (NPRCT-CU) Animal Care and Use Committee, Chulalongkorn University, Bangkok, Thailand.

## Author Contributions

KiR, AT, and WP designed all experiments. KS, BS, and KaR performed protein expression, protein purification, ELISA, and protein preparation for animal experiments. KS, SMan, SP, SS, ST, EP, CK, SB, WW, KT, and PK performed vaccination and immunogenic studies in mice. KS, SMan, SP, SS, ST, SB, WW, TK, NS, and SMal performed vaccination and immunogenic studies in non-human primates. KL performed LC-MS analysis. All authors analyzed the data and contributed to manuscript preparation.

## Conflict of Interest

WP from Chulalongkorn University is a founder/shareholder of Baiya Phytopharm Co., Ltd. BS and KR are employed by Baiya Phytopharm Co., Ltd., Thailand. WW is employed by BioNet-Asia Co., Ltd., Thailand. The remaining authors declare that the research was conducted in the absence of any commercial or financial relationships that could be construed as a potential conflict of interest.
