# Supplementary material for: Plant-Produced Receptor-Binding Domain of SARS-CoV-2 Elicits Potent Neutralizing Responses in Mice and Non-human Primates
Source: Front Plant Sci. 2021 May 13;12:682953. doi: 10.3389/fpls.2021.682953 (PMC8158422; doi:10.3389/fpls.2021.682953)

**Plant-Produced Receptor-Binding Domain of SARS-CoV-2 Elicits Potent Neutralizing Responses in Mice and Non-human Primates**

Konlavat Siriwattananon^1,2^, Suwimon Manopwisedjaroen^3^, Balamurugan Shanmugaraj^4^, Kaewta Rattanapisit^4^, Supaporn Phumiamorn^5^, Sompong Sapsutthipas^5^, Sakalin Trisiriwanich^5^, Eakachai Prompetchara^6,7^, Chutitorn Ketloy^6,7^, Supranee Buranapraditkun^6,8^, Wassana Wijagkanalan^9^, Kittipan Tharakhet^6^, Papatsara Kaewpang^6^, Kantinan Leetanasaksakul^10^, Taratorn Kemthong^11^, Nutchanat Suttisan^11^, Suchinda Malaivijitnond^11^, Kiat Ruxrungtham^6,8^, Arunee Thitithanyanont^3^, Waranyoo Phoolcharoen^1, 2^*

^1^ Research unit for Plant-produced Pharmaceuticals, Chulalongkorn University, Bangkok, Thailand

^2^ Department of Pharmacognosy and Pharmaceutical Botany, Faculty of Pharmaceutical Sciences, Chulalongkorn University, Bangkok, Thailand.

^3^ Department of Microbiology, Faculty of Science, Mahidol University, Bangkok, Thailand

^4^ Baiya Phytopharm Co., Ltd, Bangkok, Thailand

^5^ Institute of Biological Products, Department of Medical Sciences, Ministry of Public Health, Nonthaburi, Thailand

^6^ Center of Excellence in Vaccine Research and Development (Chula Vaccine Research Center, Chula VRC), Faculty of Medicine, Chulalongkorn University, Bangkok, Thailand

^7^ Department of Laboratory Medicine, Faculty of Medicine, Chulalongkorn University, Bangkok, Thailand

^8^ Department of Medicine, Faculty of Medicine, Chulalongkorn University, Bangkok, Thailand

^9^ BioNet-Asia Co., Ltd, Bangkok, Thailand

^10^ National Center for Genetic Engineering and Biotechnology (BIOTEC), National Science and Technology Development Agency, Pathum Thani, Thailand.

^11^ National Primate Research Center of Thailand-Chulalongkorn University, Saraburi, Thailand

*Correspondence: Waranyoo.P@chula.ac.th; Tel: 662-218-8359; Fax: 662-218-8357


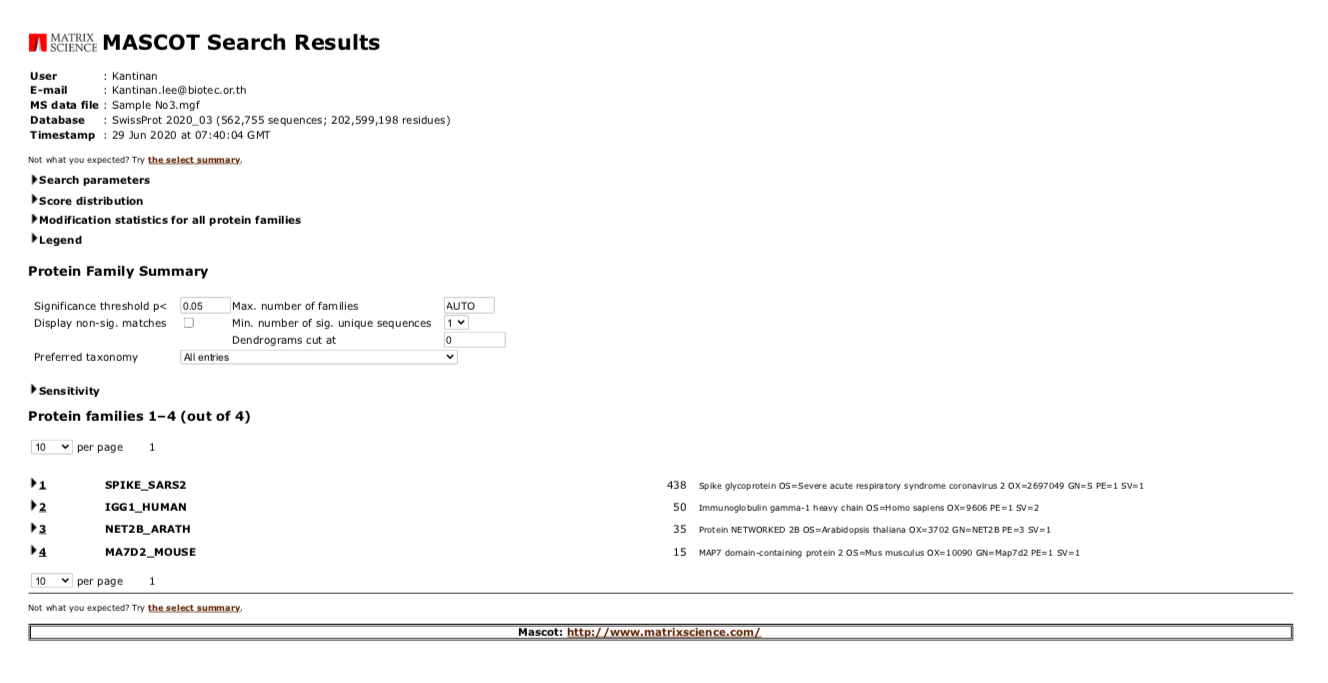

Supplement: Supplementary file 1 [file Data_Sheet_1.docx]
